# Maximising the impact of house modification with eave tubes for malaria control in Africa

**Published:** 2021-03-01

**Authors:** Bart G.J. Knols, Fredros O. Okumu

**Affiliations:** 1 Environmental Health and Ecological Sciences Department, Ifakara Health Institute, Ifakara, Tanzania;; 2 MalariaWorld, K&S Consulting, Dodewaard, The Netherlands;; 3 Soneva, Kunfunadhoo island, Maldives

## Opinion

**Figure 1.**
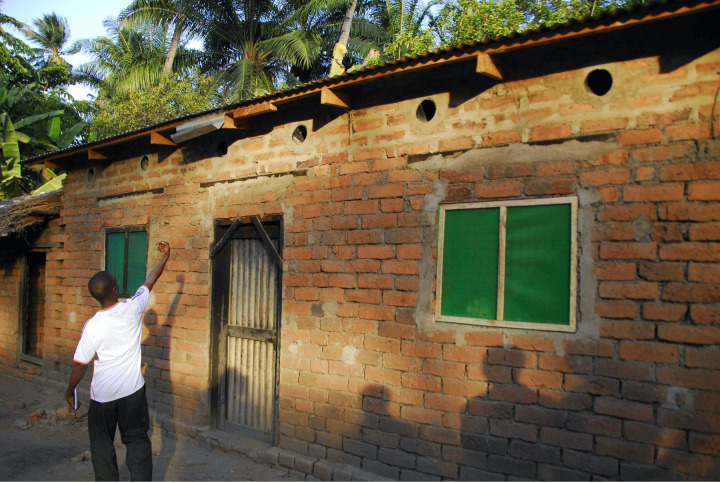
A house near Ifakara, Tanzania, fitted with window screening and eave tubes.

Insecticide-treated eave tubes represent a new tool to dramatically reduce malaria in Africa, where these were recently evaluated in a cluster ran-domised controlled trial (RCT) funded by the Bill and Melinda Gates Foundation [1]. The technology includes simple pieces of PVC piping, and their installation in otherwise closed eaves under the roof, which form the primary entry point for malaria mosquitoes into houses [2] (Figure 1). Given that the trial in Côte d’Ivoire, also included screening of windows, WHO’s Vector Control Advisory Group (VCAG) has dubbed the combined approach ‘lethal house lure’ [3]. Eave tubes, in essence, turn the entire house into a ‘lure and kill’ station, providing protection for everyone inside. Netting inserts, treated with insecticide, result in mosquitoes being killed upon contact when trying to enter the house through the tubes that funnel the indoor human-scented air outwards, luring host-seeking mosquitoes.

Results from the 2-year follow-up period in the RCT showed an impressive drop of 38% in malaria case incidence (2.29 per child-year (95% CI 1.97-2.61) in the control group and 1.43 per child-year (1.21-1.65) in the intervention group (hazard ratio 0.62, 95% CI 0.51-0.76; *p* < 0.0001)) when coverage (i.e., the proportion of houses receiving eave tubes) averaged 70% in the treatment clusters [1]. In order to become recommended by WHO as a novel vector control tool, and as such become accessible to National Malaria Control Programmes (NMCPs) through funding from the Global Fund, UNICEF or the US President’s Malaria Initiative, an additional RCT has been recommended [4].

Given the effectiveness of this technology, and its potential to provide sustained malaria prevention gains, we discuss below a potential pathway for mass uptake of the approach to achieve the highest impact on malaria possible.

Eave tubes were invented during an EU-FP7 project that brought together researchers from five different Universities and companies [5]. Initial efficacy evaluations were conducted inside large semi-field facilities at Ifakara Health Institute in Tanzania [6]. The concept built on earlier observations that African malaria mosquitoes prefer to enter houses through open eaves between the roofs and walls of houses [7]. The polyester netting initially used inside PVC tubes received an electrostatic coating charged with insecticidal particles that resulted in much-improved exposure of mosquitoes upon contacting it; to such extent that even highly pyrethroid-resistant strains of malaria mosquitoes succumbed after exposure to low doses of pyrethroids [8]. All partners in the project, including both authors of this opinion article, collectively filed a patent in order to secure the rights of exclusive exploitation of this intellectual property [9]. This patent included eave tubes but was not granted as the inventive step was not deemed significant enough because of prior knowledge in the public domain of closely related products, such as eave screens or curtains, that existed at the time and serve the same purpose. What remained was the patent relating to the insecticide-resistance breaking properties of electrostatic netting, which rests with In2Care, a Dutch company founded in 2011.

Given the fact that eave tubes are not patented technology, anyone interested in exploiting the merits thereof, can incorporate these into existing houses or those under construction. Interestingly, the EU-FP7 project team published results from semi-field studies that indicated that ‘ordinary’ pieces of insecticide-treated bed net material (Per-maNet 2.0, Vestergaard-Frandsen company) could, just like electrostatic netting, strongly impact survival of pyrethroid-susceptible mosquitoes [6]. And although resistance-breaking bed nets were still under development or had not yet received WHO pre-qualification status at the time of the EU-FP7 project (2012-2015), such materials with long-last-ing properties that far outpace the active lifespan of electrostatic netting (which is ca. 3-6 months, depending on the active ingredient) [10] have reached the market since. As such, anyone wanting to use eave tubes as a new or additional intervention today can simply use PVC pipes and cover these with pieces of multiactive (resistance-breaking) insecticidal netting material, which are now readily available. A huge advantage of bed net material is that its effective lifespan is likely to be as much as ten times that of electrostatic netting, thereby dramatically reducing the cost of the netting itself as well as servicing (i.e., re-treatment) and replacement costs. The amount of netting used for manufacturing a standard rectangular bed net (total surface area of ca. 10 m^2^) can provide enough netting to cover eave tubes in 20-25 houses (8x8 inch pieces to cover 6-inch diameter PVC tubes, yielding 200 eave tubes; each house takes 8-10 tubes). In terms of cre-ating access in an equitable and morally acceptable manner, local entrepreneurs can use PVC and existing long-lasting or resistance-breaking netting ma-terial to locally ‘manufacture’ and distribute eave tubes. Experimental hut studies in Côte d’Ivoire suggest that once the eave spaces are sealed and insecticidal tubes inserted, the need for additional window screening may be reduced [11].

The above opens perspectives for local businesses across Africa – where a wide range of piping materials, ventilation bricks, or other options exist to partially block eaves and use bed net material to cover these. Young and enthusiastic entrepreneurs could set up operations to close eave gaps and install eave tubes in houses for a small profit to drive the uptake and dissemination of this approach. To obtain the netting, they may collect and re-purpose torn nets attritioned from local homes, import insecticidal netting material with multiple actives to also kill pyrethroid-resistant mosquitoes, or set up local manufacturing facilities. Countries such as Tanzania already have local large-scale manufacturing facilities (e.g., A to Z Textiles, in Arusha), which could also provide the material. In this way eave tube technology can instantly become widely available to people who need it most in malaria-endemic countries.

Looking back at the evolution of insecticide-treated bed nets it took more than a decade after the first promising results were published [12] for nets to reach the masses in need of it. This need and should not be so long for eave tubes. For the past five years, progress in bringing down the number of malaria deaths and cases has practically stalled and every report or scientific article stresses the need for new tools. House modification efforts in-corporating eave tubes clearly present such a tool, with the added advantage that it can be readily available today to anyone in need of it and will enable application of significantly lower doses of in-secticides than currently used on bed nets or for indoor residual spraying [13]. Never again may the introduction of a new malaria vector control intervention be as easy as this one – in business terms it is called nothing more than re-purposing an exist-ing tool: the bed net.
